# PDLIM1 inhibits NF-κB-mediated inflammatory signaling by sequestering the p65 subunit of NF-κB in the cytoplasm

**DOI:** 10.1038/srep18327

**Published:** 2015-12-18

**Authors:** Rumiko Ono, Tsuneyasu Kaisho, Takashi Tanaka

**Affiliations:** 1 Laboratory for Inflammatory Regulation, RIKEN Center for Integrative Medical Sciences (IMS), RIKEN Research Center for Allergy and Immunology (RCAI), Yokohama, Kanagawa 230-0045, Japan; 2 Division of Immunobiology, Graduate School of Medical Life Science, Yokohama City University, Yokohama, Kanagawa 230-0045, Japan; 3 Department of Immunology, Institute of Advanced Medicine, Wakayama Medical University, Wakayama 641-8509, Japan; 4 Precursory Research for Embryonic Science and Technology (PRESTO), Japan Science and Technology Agency, Kawaguchi, Saitama 332-0012, Japan

## Abstract

Understanding the regulatory mechanisms for the NF-κB transcription factor is key to control inflammation. IκBα maintains NF-κB in an inactive form in the cytoplasm of unstimulated cells, whereas nuclear NF-κB in activated cells is degraded by PDLIM2, a nuclear ubiquitin E3 ligase that belongs to a LIM protein family. How NF-κB activation is negatively controlled, however, is not completely understood. Here we show that PDLIM1, another member of LIM proteins, negatively regulates NF-κB-mediated signaling in the cytoplasm. PDLIM1 sequestered p65 subunit of NF-κB in the cytoplasm and suppressed its nuclear translocation in an IκBα-independent, but α-actinin-4-dependent manner. Consistently, PDLIM1 deficiency lead to increased levels of nuclear p65 protein, and thus enhanced proinflammatory cytokine production in response to innate stimuli. These studies reveal an essential role of PDLIM1 in suppressing NF-κB activation and suggest that LIM proteins comprise a new family of negative regulators of NF-κB signaling through different mechanisms.

The transcription factor nuclear factor κB (NF-κB) is well known to play an important role in inflammatory reaction^1^,^2^. NF-κB consists of five members, p65 (RelA), NF-κB1 (p50/p105), NF-κB2 (p52/p100), c-Rel and RelB, which form various homo- and heterodimers. In the steady state, NF-κB is associated with members of the inhibitor of κB (IκB) protein family, which includes IκBα, and sequestered in the cytoplasm where it has no known activity. Innate immune cells, such as macrophages and dendritic cells, detect invading microbial pathogens, including bacteria and viruses, by their pathogen sensors (e.g. Toll-like receptors [TLR]). Signal through TLR leads to phosphorylation of IκB proteins and consequently their degradation by the proteasome. Heterodimers of p65 and p50 then translocate to the nucleus, bind to κB sites in the promoters/enhancers of target genes, including proinflammatory cytokines such as interleukin-6 (IL-6) and IL-12, and induce their transcription^1^,^2^. Although these responses are critical for eliminating invading pathogens, excessive activation of NF-κB may cause inflammatory and autoimmune human diseases, such as asthma and arthritis^3^,^4^. However, the molecular mechanisms that negatively regulate these responses are not completely understood.

We previously reported that PDLIM2 (also known as SLIM or mystique) negatively regulates NF-κB activation. PDLIM2 is a nuclear protein containing both PDZ (postsynaptic density 65-discs large-zonula occludens 1) and LIM (abnormal cell lineage 11-islet 1-mechanosensory abnormal 3) domains and belongs to a large family of LIM proteins^5^,^6^,^7^. The LIM domain of PDLIM2 has a ubiquitin E3 ligase activity, and PDLIM2 acts as a nuclear ubiquitin ligase, catalyzing polyubiquitination of the p65 subunit of NF-κB. PDLIM2 binds to p65 and promotes p65 polyubiquitination through its LIM domain. In addition, PDLIM2 targets p65 to discrete intranuclear compartments called Promyelocytic Leukemia (PML) nuclear bodies, through PDZ domain. Polyubiquitinated p65 is ultimately degraded by proteasomes in these compartments. Consistent with this mechanism of action, PDLIM2 deficiency results in increased amounts of nuclear p65 and augmented production of proinflammatory cytokines by dendritic cells^8^. To date, more than 30 proteins containing a LIM domain, have been classified as members of the LIM protein family^9^, which is divided into subgroup based on domain structure. Ten proteins that have both PDZ and LIM domains, PDLIM1, PDLIM2, PDLIM3/ALP, PDLIM4/Ril, PDLIM5/ENH, PDLIM6/ZASP/Cypher, PDLIM7/Enigma, LIMK1, LIMK2 and LMO7 are included in the PDZ-LIM protein subfamily. Since both PDZ and LIM domains are involved in protein-protein interactions, previous studies of these molecules have been focused on the identification of their binding partners, and have also shown that they are implicated in the regulation of various biological processes, including cytoskeleton organization and oncogenesis^10^. However, their functions in the immune system remain completely unknown. We have therefore investigated the roles of PDZ-LIM protein family members in the regulation of immune function.

Here we demonstrate that PDLIM1 (also known as CLP36 or Elfin)^11^,^12^, is a cytoplasmic LIM protein that negatively regulates NF-κB-mediated signaling in dendritic cells but through a different mechanism from PDLIM2. PDLIM1 bound to and sequestered p65 in the cytoplasm possibly by interaction with the actin binding protein α-actinin, and suppressed its nuclear translocation of p65 protein. Notably, the activity of PDLIM1 to retain p65 in the cytoplasm was IκBα-independent. PDLIM1-deficient dendritic cells produced more proinflammatory cytokines than wild-type cells in response to TLR stimulation. Our work suggests that the PDZ-LIM protein family consists of novel negative regulators of NF-κB-mediated inflammatory responses.

## Results

### PDLIM1 is a cytoplasmic protein expressed in dendritic cells

**Figure 1:**
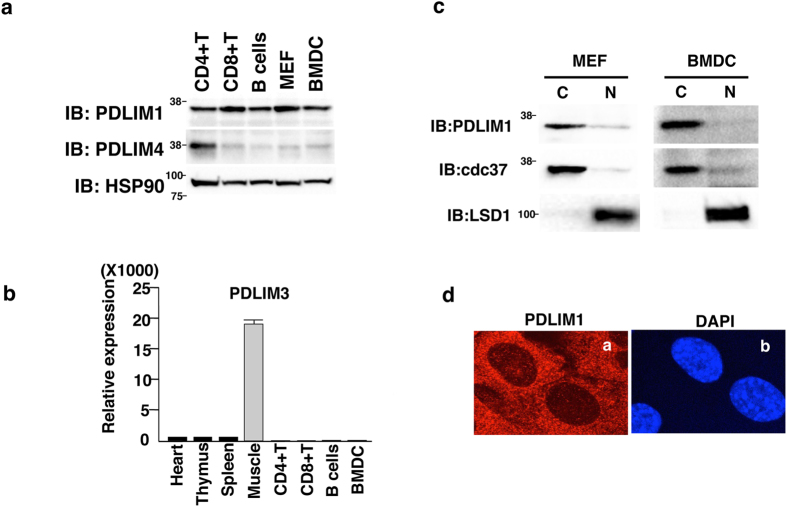
PDLIM1 is a cytoplasmic protein expressed in dendritic cells. (**a**) Western blot analysis of PDLIM1 and PDLIM4 expression in primary immune cells. Whole cell lysates were subjected to immunoblot (IB) with anti-PDLIM1, PDLIM4 and HSP90 antibodies. Western blots are representative of at least three independent experiments. (**b**) Real-time RT-PCR analysis of PDLIM3 expression in mouse tissues and primary immune cells. Data are means ± SD from at least three independent experiments. (**c**) Bone-marrow derived dendritic cells (BMDC) and mouse embryonic fibroblasts (MEF) were fractionated into cytoplasmic (C) and nuclear (N) fractions, which were then analyzed by Western blotting with anti-PDLIM1 antibody. The purity of the fractionations was confirmed by anti-cdc37 (cytoplasm) or anti-LSD1 (nucleus) antibody. Western blots are representative of at least three independent experiments. (**d**) Cytoplasmic localization of PDLIM1 in MEF was determined by indirect immunofluorescence with an *in situ* proximity-ligation assay. DAPI, nuclear staining. Original magnification, x630. Data are representative of at least three independent experiments.

In this study, we have sought to identify the PDZ-LIM proteins, in addition to PDLIM2, that are critically involved in the negative regulation of NF-κB signaling in dendritic cells. Among the ten PDZ-LIM proteins, PDLIM1, PDLIM3 and PDLIM4 are most closely related to PDLIM2, since they all contain one PDZ domain and one LIM domain, whereas other PDZ-LIM proteins have one PDZ domain plus three LIM domains or other conserved domains^10^. We therefore focused on PDLIM1, 3 and 4 and examined their expression in dendritic cells. We have previously shown that PDLIM2 is ubiquitously expressed in immune cells^6^. We found that PDLIM1 is also highly expressed in all immune cells tested, including dendritic cells, while PDLIM4 is exclusively expressed in CD4^+^T cells, but not in dendritic cells (Fig. 1a). On the other hands, the expression of PDLIM3 has been reported to be primarily expressed in muscle cells^13^. We too confirmed that PDLIM3 expression was barely detectable in immune cells, including dendritic cells (Fig. 1b). These observations prompted us to select PDLIM1 to further investigate the roles of PDZ-LIM proteins in the regulation of NF-κB signaling in dendritic cells. PDLIM1 was thought to be an adaptor protein interacting α-actinin, an actin binding protein, and previous studies have shown the roles of PDLIM1 in the formation of actin stress fibers^12^. It, however, remains completely unclear how PDLIM1 functions in the immune system. We first examined subcellular localization of PDLIM1 in dendritic cells and fibroblasts. In contrast to the nuclear expression of PDLIM2, PDLIM1 was predominantly located in the cytoplasm in both of these cell types (Fig. 1c). Cytoplasmic localization of PDLIM1 in fibroblasts was further confirmed by immunofluorescent staining (Fig. 1d).

### PDLIM1 negatively regulates NF-κB signaling

**Figure 2:**
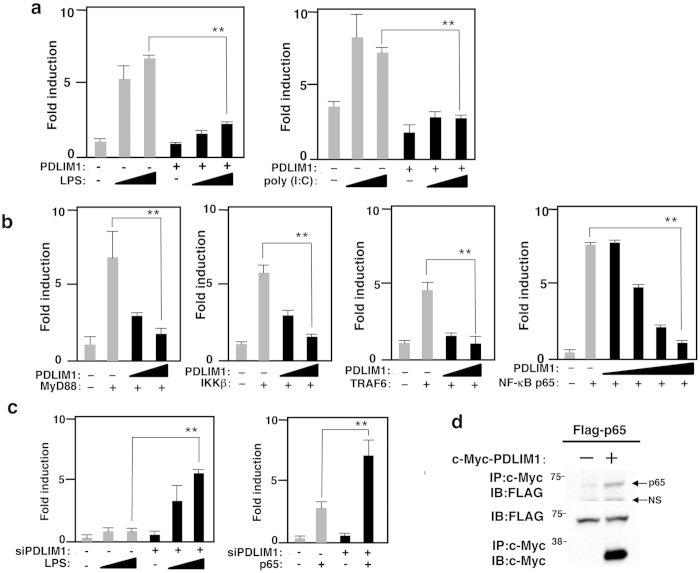
PDLIM1 binds to p65 and suppresses NF-κB signaling. (**a**) Luciferase activity in MEF transfected with a pGL4-NF-κB luciferase reporter construct along with or without a plasmid encoding PDLIM1, then left untreated or treated with LPS (2 and 10 ng/ml) or poly(I:C) (10 and 50 μg/ml) for 5 hr. Data are representative of at least three independent experiments. (**b**) Luciferase activity in 293T cells transfected with an ELΑΜ−1 luciferase reporter construct (ELAM-1-luc) with or without plasmids encoding MyD88, TRAF6, IKKβ or p65 in absence or presence of increasing amounts (wedge) of PDLIM1. (**c**) Luciferase activity in MEF first transfected with control or PDLIM1-specific siRNA and then with a ELΑΜ−1 luciferase reporter construct, which were then stimulated without or with LPS for 6 hours (left), or MEF first transfected with control or PDLIM1-specific siRNA and then with a ELΑΜ−1 luciferase reporter construct without or with the plasmid encoding p65 (right). Data are representative of at least three independent experiments and are shown as means ± SD. **P < 0.01. (**d**) PDLIM1 interacts with the p65 subunit of NF-κB. 293T cells were transfected with a FLAG-p65 expression plasmid along with or without PDLIM1. The lower bands in top blot are non-specific bands (NS). Whole cell extracts were immunoprecipitated with anti-c-Myc, and immunoblotted with anti-FLAG. Western blots are representative of at least three independent experiments.

Since PDLIM2 negatively regulates TLR-mediated NF-κB signaling, we next examined the effect of PDLIM1 on TLR-induced, NF-κB-mediated gene activation in a reporter assay. Mouse embryonic fibroblasts (MEF) were transfected with a plasmid encoding a luciferase regulated by NF-κB. Twenty hours after transfection, cells were left untreated or treated with the TLR ligands, LPS or poly(I:C), for 5 hr, and then assayed for luciferase activity. Stimulation of cells with either TLR ligands augmented luciferase reporter activity, whereas coexpression of PDLIM1 potently suppressed this TLR-induced luciferase reporter transactivation (Fig. 2a), Since PDLIM1 is a cytoplasmic protein, we predicted that it might interact with cytoplasmic signaling molecules to suppress TLR-induced signaling. We therefore cotransfected 293T cells with MyD88, TRAF6, IKKβ or p65, to activate the NF-κB luciferase construct, together with or without PDLIM1 to test if PDLIM1 could inhibit gene activation by any of these molecules. PDLIM1 markedly inhibited MyD88-, TRAF6-, IKKβ-, and p65-induced NF-κB-mediated transactivation of the reporter in a dose-dependent manner (Fig. 2b). These data suggested that p65, the most downstream molecule in this panel, was likely to be the target of PDLIM1. Moreover, specific knockdown of PDLIM1 by small interfering RNA (siRNA) resulted in a substantial enhancement of LPS-induced, or p65-mediated NF-κB transactivation (Fig. 2c). We then examined if PDLIM1 bound to the p65 subunit of NF-κB. 293T cells were transiently transfected with expression plasmids encoding c-Myc-tagged PDLIM1 with or without FLAG-tagged p65. PDLIM1 was immunoprecipitated with p65 only when PDLIM1 and p65 were coexpressed (Fig. 2d).

### PDLIM1 inhibits NF-κB-mediated inflammatory responses

**Figure 3:**
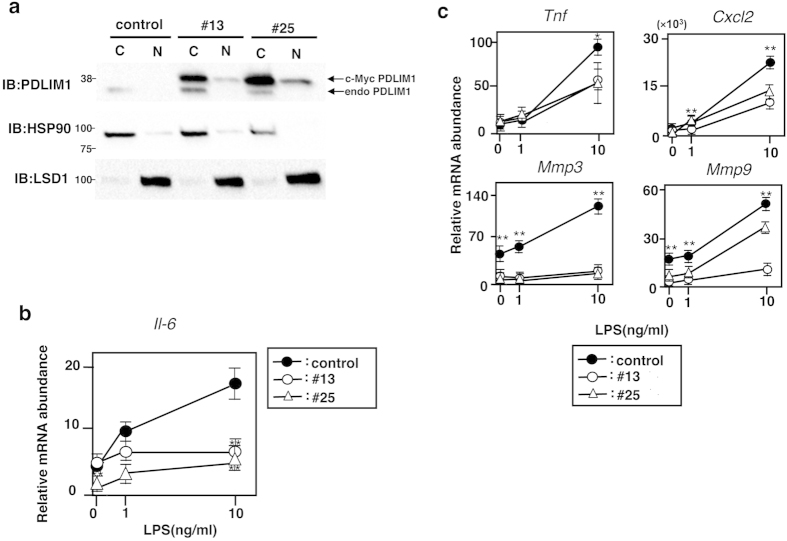
PDLIM1 inhibits NF-κB-mediated gene expression. (**a**) Cytoplasmic and nuclear extracts from NIH3T3 cells clones, stably expressing Myc-PDLIM1 (#13, #25) or empty vector (control) were immunoblotted with anti-PDLIM1. Upper bands correspond to stably expressed c-Myc-tagged PDLIM1 and lower bands are endogenous PDLIM1. The purity of the fractionations was confirmed by blotting with anti-HSP90 (cytoplasm) or anti-LSD1 (nucleus). Western blots are representative of at least three independent experiments. (**b**) Real-time RT-PCR analysis of IL-6 in control NIH3T3 cells and NIH3T3 cells expressing PDLIM1 (#13, #25) stimulated for 2 h with LPS. Data are means ± SD from at least three independent experiments. **P < 0.01. (**c**) Real-time RT-PCR analysis of LPS-inducible genes in control NIH3T3 cells and NIH3T3 cells expressing PDLIM1 (#13, #25), unstimulated or stimulated with LPS for 2 h. Data are means ± SD from at least three independent experiments. *P < 0.05; **P < 0.01.

We next examined whether PDLIM1 could affect the expression of the endogenous NF-κB target gene. NIH3T3 cells were stably transfected with a PDLIM1 expression plasmid or vector alone as control, and two clones that exhibited high level of PDLIM1 expression were established (Fig. 3a). As shown in Fig. 3b, LPS-induced IL-6 expression was markedly impaired in the two independent NIH3T3 clones that overexpressed PDLIM1. Moreover, we selected a subset of TLR-dependent genes whose expression is increased by LPS stimulation in fibroblasts, and investigated their expression in control and NIH3T3 cells expressing PDLIM1. We found that LPS-induced expression of cytokine (TNFα), chemokine (CXCL2) and matrix metalloproteinases (MMP3 and MMP9) was also impaired in an NIH3T3 clone expressing PDLIM1 (Fig. 3c). These data suggested that PDLIM1 is a negative regulator of NF-κB-mediated inflammatory signaling.

### PDLIM1 suppresses LPS-induced nuclear translocation of p65

**Figure 4:**
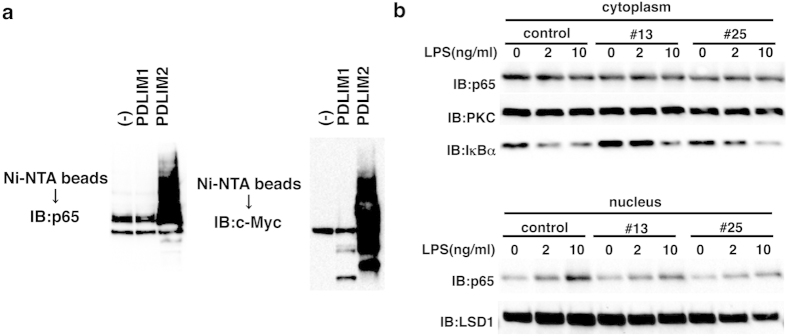
PDLIM1 suppresses LPS-induced nuclear translocation of p65. (**a**) Ubiquitination assay for p65 in 293T cells transfected with plasmids encoding histidine-tagged ubiquitin, p65 and PDLIM1, PDLIM2 or empty vector (−). Ubiquitinated proteins were purified with Ni-NTA beads. Polyubiquitination of p65 (left) or autoubiquitination of PDLIM1/PDLIM2 (right) were analyzed by immunoblot with anti-p65 or anti-c-Myc antibody, respectively. Western blots are representative of at least three independent experiments. (**b**) Immunoblot of cytoplasmic and nuclear extracts of control NIH3T3 cells (control) and NIH3T3 clones expressing PDLIM1 (#13, #25), either untreated or treated for 1 h with LPS (2 and 10 ng/ml), analyzed with the indicated antibodies (IB, left margin). Western blots are representative of at least three independent experiments.

We next investigated the mechanisms by which PDLIM1 negatively regulated TLR-induced p65 activation. Since PDLIM2 is an ubiquitin E3 ligase targeting p65 for proteasome-dependent degradation, we examined if PDLIM1 also promote ubiquitination and degradation of p65. As shown in Fig. 4a, p65 was polyubiquitinated when it was coexpressed with PDLIM2. However, PDLIM1 expression did not lead to the ubiquitination of either p65 or PDLIM1 itself, suggesting that PDLIM1 does not have E3 ubiquitin ligase activity. Consistently, overexpression of PDLIM1 did not promote degradation of p65 in both cytoplasmic and nuclear compartments (data not shown). We then examined whether PDLIM1 affects the nuclear translocation of p65. Cytoplasmic and nuclear extracts of control NIH3T3 cells and NIH3T3 clones expressing PDLIM1, either left untreated or treated with LPS for 1 hr, were prepared, followed by Western blot analysis. Nuclear translocation of p65 was markedly decreased in NIH3T3 clones expressing PDLIM1 compared to control cells (Fig. 4b). Meanwhile, LPS-induced degradation of IκBα was normal in NIH3T3 clones expressing PDLIM1 (Fig. 4b), indicating that TLR signaling leading to IκBα degradation, the last step to activate NF-κB in the cytoplasm, was not impaired in these cells. Considering the cytoplasmic localization of PDLIM1 (Fig. 3a), these data suggest that PDLIM1 directly inhibits nuclear translocation of p65 without affecting upstream signaling events.

### PDLIM1 sequesters p65 in the cytoplasm via association with α-actinin-4

**Figure 5:**
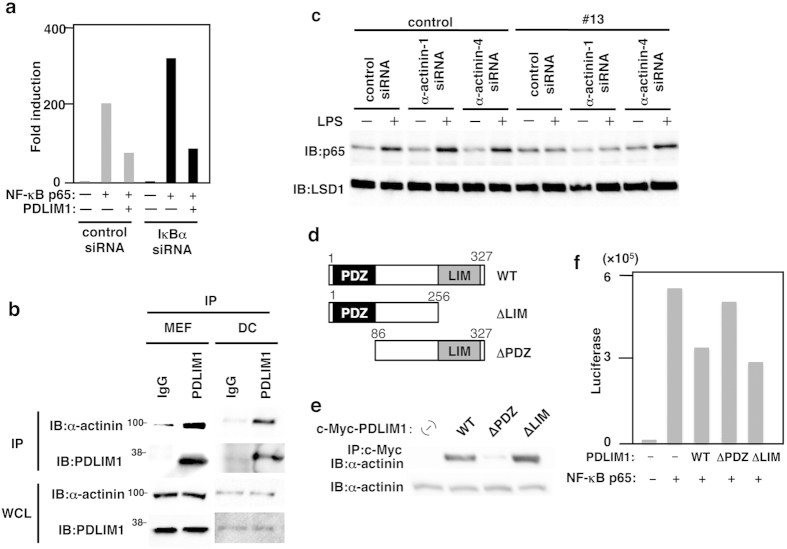
PDLIM1 sequesters p65 in the cytoplasm via association with α-actinin-4. (**a**) Luciferase activity in 293T cells transfected first with control siRNA or IκBα-specific siRNA, then transfected with the ELAM-1-luciferase reporter construct along with expression plasmids encoding p65 with or without PDLIM1. Data are representative of at least three independent experiments. (**b**) Endogenous interaction between PDLIM1 and α-actinin in MEF (left) and DC (right). Whole cell lysates were immunoprecipitated with anti-PDLIM1 and immunoblotted with anti-α-actinin or anti-PDLIM1 antibody. Western blots are representative of at least three independent experiments. (**c**) Nuclear extracts in control NIH3T3 cells and NIH3T3 cells expressing PDLIM1, transfected with control, α-actinin-1-specific or α-actinin-4-specific siRNA and left untreated or treated with LPS for 1 hr, analyzed by immunoblot with the indicated antibodies (IB, left margin). Western blots are representative of at least three independent experiments. (**d**) Depiction of wild-type PDLIM1 (WT) and PDLIM1 mutants lacking the PDZ domain (∆PDZ) or LIM domain (∆LIM). (**e**) Identification of the domain of PDLIM1 that is required for its association with α-actinin. Whole cell lysates in 293T cells transfected with wild-type or mutants PDLIM1 were immunoprecipitated with anti-c-Myc and immunoblotted with anti-α-actinin antibody. Western blots are representative of at least three independent experiments. (**f**) Effects of PDLIM1 mutants on p65-mediated gene activation in 293T cells transfected with the ELAM-1-luciferase reporter construct plus expression plasmids encoding p65 and wild-type or PDLIM1 mutants, assessed by measurement of luciferase activity. Data are representative of at least three independent experiments.

We further investigated the molecular mechanisms by which PDLIM1 suppressed nuclear translocation of p65. IκBα has been thought to be the major component to regulate cytoplasmic retention of NF-κB^1^. We therefore examined whether IκBα was involved in PDLIM1-mediated suppression of NF-κB signaling. 293T cells were first transfected with control siRNA or IκBα-specific siRNA, and then transfected with the luciferase reporter containing NF-κB binding sites in the absence or presence of PDLIM1. PDLIM1 could inhibit p65-induced NF-κB-mediated transactivation even in cells lacking IκBα expression, indicating that the activity of PDLIM1 is IκBα-independent (Fig. 5a). The α-actinin family consists of four structurally related actin binding proteins, non-muscle α-actinin-1 and α-actinin-4, and muscle α-actinin-2 and α-actinin-3^14^. PDLIM1 has been reported to be associated with α-actinin-1 and α-actinin-4 through its PDZ domain and localized to actin stress fibers in the cytoplasm^12^. We next determined whether α-actinin was involved in PDLIM1-mediated suppression of p65 nuclear translocation. We first examined the endogenous association of PDLIM1 with α-actinin in fibroblasts (MEF) and dendritic cells (DC), and found that the two proteins could be co-immunoprecipitated in both types of cells (Fig. 5b). We then examined the roles of α-actinin in the ability of PDLIM1 to suppress nuclear translocation of p65. Control and NIH3T3 cells expressing PDLIM1 were transfected with control, α-actinin-1-specific or α-actinin-4-specific siRNA and left untreated or treated with LPS for 1 hr. Cytoplasmic and nuclear extracts were prepared and analyzed by immunoblot with anti-p65. Specific knockdown of α-actinin-4, but not α-actinin-1, reverted PDLIM1-mediated suppression of nuclear translocation of p65 in NIH3T3 cells expressing PDLIM1 (Fig. 5c). To clarify the function of individual domains in PDLIM1-mediated cytoplasmic sequestration of p65, we next generated PDLIM1 mutants lacking the PDZ domain (∆PDZ-PDLIM1) or the LIM domain (∆LIM-PDLIM1) (Fig. 5d). Consistent with previous reports^12^, wild-type and ∆LIM-PDLIM1 could still associate with α-actinin, whereas ∆PDZ-PDLIM1 could not (Fig. 5e). We then assessed the ability of these mutants to inhibit NF-κB activation in the luciferase reporter assay. As shown in Fig. 5f, ∆LIM-PDLIM1 retained its inhibitory activity at a level essentially equal to wild-type PDLIM1, whereas ∆PDZ-PDLIM1 was impaired in suppression of p65-mediated gene activation, suggesting that association of PDLIM1 with α-actinin through PDZ domain was essential for PDLIM1 to inhibit NF-κB signaling. Taking these data together, we concluded that PDLIM1 sequesters p65 in the cytoplasm and suppresses its nuclear translocation to inhibit inflammatory signaling in an IκBα-independent but α-actinin-4-dependent manner.

### p65-mediated inflammatory responses are enhanced in *Pdlim1* ^*−/−*^ dendritic cells

**Figure 6:**
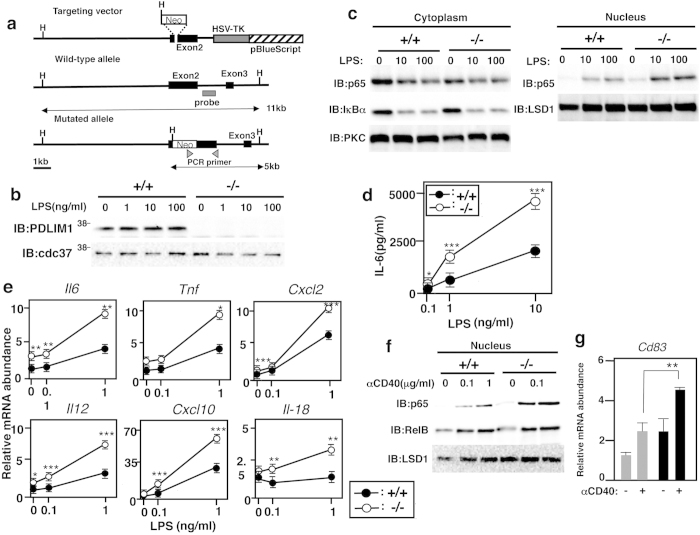
Enhanced p65-mediated inflammatory responses in *Pdlim1*^−/−^ dendritic cells. (**a**) Schematic diagram of the *Pdlim1* targeting vector, wild-type allele and mutated allele for generation of PDLIM1-deficient mice. The arrowheads represent the primers used for screening by PCR analysis and the probe used for Southern blot analysis is shown as a closed bar. Abbreviation for restriction sites is as follows: H, *HindIII*. The two headed arrows indicate the size of the *HindIII* fragment in the WT and mutant allele. (**b**) Western blot analysis of PDLIM1 expression in cytoplasmic extracts of bone marrow-derived dendritic cells from *Pdlim1*^+/+^ and *Pdlim1*^−/−^ mice, either untreated or treated for 1 h with LPS. Western blots are representative of at least three independent experiments. (**c**) Immunoblot of cytoplasmic and nuclear extracts of *Pdlim1*^+/+^ and *Pdlim1*^−/−^ dendritic cells, either untreated or treated for 1 h with LPS (10 and 100   ng/ml), analyzed with the indicated antibodies (IB, left margin). Western blots are representative of at least three independent experiments. (**d**) Enzyme-linked immunosorbent assay (ELISA) of IL-6 in culture supernatants of *Pdlim1*^+/+^ and *Pdlim1*^−/−^ dendritic cells, stimulated for 24 h with the indicated concentrations of LPS. Data are means ± SD from at least three independent experiments. *P < 0.05; ***P < 0.001. (**e**) Real-time RT-PCR analysis of LPS-inducible genes in *Pdlim1*^+/+^ and *Pdlim1*^−/−^ dendritic cells, unstimulated or stimulated with LPS for 2 h. Data are means ± SD from at least three independent experiments. *P < 0.05; **P < 0.01; ***P < 0.001. (**f**) Immunoblot of nuclear extracts of *Pdlim1*^+/+^ and *Pdlim1*^−/−^ B cells, either untreated or treated for 1 h with anti-CD40 (0.1 and 1 μg/ml), analyzed with the indicated antibodies (IB, left margin). Western blots are representative of two independent experiments using *Pdlim1*^+/+^ (N = 7) and *Pdlim1*^−/−^ (N = 7) mice. (**g**) Real-time RT-PCR analysis of CD83 in *Pdlim1*^+/+^ and *Pdlim1*^−/−^ B cells, stimulated for 2 h with anti-CD40 antibody (0.1 μg/ml). Data are means ± SD (N = 3). **P < 0.01.

Finally, to investigate the roles of PDLIM1 *in vivo*, we generated PDLIM1 deficient mice by gene targeting (Fig. 6a). The *Pdlim1*^−/−^ mice were born at the expected Mendelian frequency and appeared healthy. Western blot analysis of dendritic cells confirmed that PDLIM1 protein was undetectable in the *Pdlim1*^−/−^ cells (Fig. 6b). PDLIM1-deficient mice had normal numbers of immune cells, including CD4^+^ T cells, CD8^+^ T cells, B cells, macrophages and dendritic cells.

To examine the effects of PDLIM1 deficiency on TLR-mediated p65 activation in dendritic cells, bone marrow-derived dendritic cells from *Pdlim1*^+/+^ and *Pdlim1*^−/−^ mice were stimulated with LPS and analyzed by immunoblot with anti-p65. There was less cytoplasmic and more nuclear p65 in *Pdlim1*^−/−^ cells than LPS-stimulated *Pdlim1*^+/+^ cells (Fig. 6c). Notably, significant amounts of p65 were already present in the nuclei of *Pdlim1*^−/−^ cells even without LPS stimulation. On the other hands, LPS-stimulated degradation of IκBα in *Pdlim1*^+/+^ and *Pdlim1*^−/−^ cells was comparable (Fig. 6c). Since PDLIM1 is in the cytoplasm and PDLIM1 does not promote p65 degradation, the increase in nuclear p65 in *Pdlim1*^−/−^ dendritic cells was likely to be due to its enhanced translocation, rather than to decreased degradation of p65 in the nucleus.

Next, we examined TLR-induced proinflammatory cytokine production by *Pdlim1*^−/−^ dendritic cells. Consistent with our finding in PDLIM1-expressing NIH3T3 cells, *Pdlim1*^−/−^ dendritic cells produced two- to threefold more IL-6 in response to LPS than the *Pdlim1*^+/+^ cells (Fig. 6d). We further selected a subset of TLR-dependent genes whose expression is increased by LPS stimulation in dendritic cells, and analyzed their expression in *Pdlim1*^+/+^ and *Pdlim1*^−/−^ dendritic cells by real-time PCR. As shown in Fig. 6e, the expression of all genes tested was upregulated in *Pdlim1*^−/−^ dendritic cells compared to *Pdlim1*^+/+^ cells. These data suggest that PDLIM1 inhibits NF-κB-mediated inflammatory responses by suppressing nuclear translocation of p65 protein. We also analyzed NF-κB signaling in B cells. We purified resting B cells from *Pdlim1*^+/+^ and *Pdlim1*^−/−^ mice and stimulated them with anti-CD40 antibody. We then checked nuclear translocation of p65 and RelB and found that nuclear translocation of p65, but not RelB was enhanced in anti-CD40-stimulated *Pdlim1*^−/−^ B cells, compared to wild-type cells (Fig. 6f), suggesting that PDLIM1 is not a general inhibitor for NF-κB proteins, targeting p65, but not RelB, for inactivation. Moreover, anti-CD40-induced expression of CD83 was also enhanced in *Pdlim1*^−/−^ B cells, compared to wild-type cells (Fig. 6g). These data suggest that PDLIM1 negatively regulates p65-mediated NF-κB signaling also in B cells.

## Discussion

Dysregulated activation of NF-κB is involved in the pathogenesis of human autoimmune and allergic diseases^1^,^2^, thus the activation of this signal transduction pathway should be strictly regulated at multiple levels to prevent immunopathology. Several factors directly target NF-κB for inactivation by different mechanisms. These include IκBNS^15^, Ahr (aryl hydrocarbon receptor)^16^, Bcl-3 (B cell leukemia/lymphoma 3)^17^, and Nurr1 (nuclear receptor related 1)^18^, which are transcriptional repressors, as well as COMMD1 (COMM domain containing 1)^19^, which promotes p65 degradation as a ubiquitin E3 ligase, and PIAS1/3^20^,^21^, which block the DNA binding activity of p65. However, the major mechanism to regulate NF-κB activity is to control the intracellular localization of the p65/p50 heterodimer, a function performed mainly by IκB proteins. In the steady state, three classical IκBs, IκBα, IκBβ and IκBε, bind to and sequester NF-κB in the cytoplasm by masking a conserved nuclear localization sequence (NLS) in the Rel-homology domain (RHD) of NF-κB, thereby attenuating nuclear translocation of NF-κB and subsequent gene activation^1^. In the present study, we identify a novel factor essential for regulating p65 nuclear translocation. PDLIM1 sequesters p65 in the cytoplasm and inhibits its nuclear translocation, thereby attenuating NF-κB-mediated inflammatory signaling. Notably, PDLIM1 can suppress NF-κB-mediated gene activation in a luciferase assay even in the absence of IκBα. Moreover, the amount of nuclear p65 in LPS-stimulated PDLIM1-deficient dendritic cells was increased compared to that in wild-type cells, whereas the degradation of IκBα in the cytoplasm occurred normally in these cells. Our data clearly show that the activity of PDLIM1 to sequester p65 in the cytoplasm is IκBα–independent. In addition to undergoing LPS-induced nuclear translocation, NF-κB continuously shuttles between cytoplasm and nucleus even in the absence of cellular stimulation, since the effect of IκBα to mask the NF-κB NLS is only partially effective and IκBα itself contains a nuclear export sequence (NES)^1^. We hypothesize that PDLIM1 may bind to such shuttling p65 and thereby negatively regulate the background level of NF-κB-mediated inflammatory signaling in unstimulated cells. This hypothesis is supported by our findings that the amount of nuclear p65 and the expression of proinflammatory cytokines were increased in *Pdlim1*^−/−^ dendritic cells compared to *Pdlim1*^+/+^ cells even without LPS stimulation, probably due to enhanced shuttling of p65 into the nucleus in the absence of PDLIM1.

We also demonstrated that PDLIM1-mediated cytoplasmic sequestration of p65 depends on α-actinin-4. Mechanistically, PDLIM1 binds to α-actinin through its PDZ domain and suppresses p65-mediated gene activation in a PDZ domain-dependent manner. Previous reports showed that PDLIM1 is localized to actin stress fiber in the cytoplasm through its PDZ domain^12^. Notably, disruption of the actin cytoskeleton has been reported to increase NF-κB-dependent transcriptional activity and cytokine production^22^,^23^, suggesting that actin stress fibers contribute to the inhibition of NF-κB signaling. Moreover, it was also reported that the actin cytoskeleton negatively regulates T-cell receptor (TCR)-mediated cytokine production^24^. Based on all of these observations, we propose a novel mechanism to control nuclear translocation of NF-κB. PDLIM1 retains p65 by association with actin stress fibers in the cytoplasm, likely by interaction with the fibers by binding to α-actinin in a PDZ domain-dependent fashion. The PDZ domain is a protein-protein interaction module that can bind to a variety of intracellular components, including enzymes, receptors and the cytoskeleton^25^. It is known that the PDZ domains of other PDZ-LIM proteins also associate with α-actinin and are involved in transporting cell signaling molecules to the appropriate intracellular locations^13^,^26^,^27^,^28^. For example, PDLIM2 binds to both α-actinin and p65 and transports nuclear p65 into discrete intranuclear compartments in a PDZ domain-dependent manner, facilitating its proteasomal degradation in these compartments. In neuronal cells, PDLIM4 binds to AMPA glutamate receptors through the LIM domain, and to α-actinin through the PDZ domain, thereby transporting AMPA receptors to dendrite spines by a mechanism dependent on α-actinin and actin^29^. Taken together, we speculate that PDZ-LIM proteins may act as adaptors between the cytoskeleton and intracellular signaling components, thereby regulating diverse cell signaling pathways.

Although we have not yet clarified how PDLIM1 activity itself is regulated, we expect that the activity of PDLIM1 to retain p65 in the cytoplasm will be down-regulated when cells are stimulated, and p65 would then be released and translocated to the nucleus. Since PDLIM1 is constitutively expressed in dendritic cells and its abundance is not altered in response to LPS, its activity is unlikely to be regulated at a transcriptional or translational level. Notably, our immunofluorescent staining experiments showed that a certain percentage of PDLIM1 entered the nucleus in response to LPS stimulation (data not shown). Previous studies also demonstrated that some PDLIM1 could be detected in the nucleus and this localization was more pronounced with a PDLIM1 mutant lacking the PDZ domain^12^,^30^. Moreover, it was also reported that α-actinin-4 can be translocated into the nucleus^14^. Taken together, we speculate that the association between PDLIM1 and actin stress fibers might be disrupted in response to LPS stimulation, which allows p65 to translocate into the nucleus together with or without PDLIM1 and/or α-actinin-4. Although further experiments will be needed to test this model, the activity of PDLIM1 might be regulated by post-translational modification, such as phosphorylation, a possibility supported by the previous reports showing that the activity of PDLIM2 and PDLIM4 can be regulated by protein kinase C (PKC)- or PKA-mediated serine phosphorylation, respectively^31^,^32^.

In this study, we demonstrate that PDLIM1 deficiency in mice results in augmented production of proinflammatory cytokines and chemokines, including IL-6, IL-12, TNFα, IL-18, CXCL2 and CXCL10, by dendritic cells, indicating that the PDLIM1 negatively regulates NF-κB-mediated inflammatory innate immune responses. Constitutive activation of NF-κB at sites of inflammation is observed in certain human autoimmune and inflammatory diseases, such as rheumatoid arthritis and bronchial asthma^3^. Thus, the PDLIM1-mediated pathway to inhibit p65 activation could be a useful new molecular target for the treatment of autoimmune and inflammatory diseases.

## Materials and Methods

### Expression vectors

C-Myc-tagged PDLIM1 was generated by subcloning of the coding sequence of mouse *Pdlim1* into pCMV-Myc (Clontech). For ∆LIM and ∆PDZ mutants, the cDNA corresponding to amino acids 2–256 or 86–327, respectively, of *Pdlim1* was subcloned into pCMV-Myc. C-Myc-tagged PDLIM2 and FLAG-tagged p65 were previously described^6^,^8^. The murine TRAF6 and MyD88 expression vectors (pUNO-mTRAF6 and pUNO-mMyD88) were purchased from InvivoGen. The plasmid expressing IKKβ was generated by subcloning of the coding sequence of mouse *Ikk*β into pEF-DEST51 (Invitrogen). For generating stable transformants of NIH3T3 cells, mouse *Pdlim1* was subcloned into pCAG-Neo (Wako) (pCAG-PDLIM1). The ELAM-1 luciferase reporter construct was kindly provided by D. Golenbock. The pGL4.32-NF-κB luciferase construct (luc2P/NF-κB-RE/Hygro) was purchased from Promega.

### Reagents and antibodies

LPS (from *Salmonella enterica*; L-2262) was purchased from Sigma. Poly (I:C) was purchased from InvivoGen. Human ligand for the receptor tyrosine kinase Flt3 (Flt3L) was from Peprotech. Anti-CD40 antibody (#102907) was purchased from BioLegend. Anti-DYKDDDDK (NU01102) antibody was purchased from Nacalai USA and used as anti-FLAG antibody. Anti-p65 (sc-372), IκBα (sc-371), HSP90 (sc-7947), protein kinase C (PKC; sc-10800) antibodies were purchased from Santa Cruz Biotechnology. Anti-LSD1 (#2184) and anti-RelB (#4922) antibodies were purchased from Cell Signaling Technology. Anti-Cdc37 (MA3-029) antibody was purchased from Affinity BioReagents. Anti-α-actinin (A-5044) antibody was purchased from Sigma. Anti-c-Myc (M047-3) antibody and anti-c-Myc antibody-conjugated agarose (M047-8) were purchased from MBL. Anti-PDLIM1 (ab129015) antibody was purchased from Abcam. The secondary antibodies HRP-goat anti-rabbit IgG (656120) was purchased from Zymed and HRP-conjugated sheep anti-mouse IgG (NA931) was from GE Healthcare.

### Cells, transfection, reporter assay

Mouse embryonic fibroblasts (MEF) prepared from 13.5 dpc embryos and 293T cells were cultured in DMEM supplemented with 10% fetal bovine serum (FBS). CD4^+^, CD8^+^, CD19^+^ and CD11c^+^ cells were purified from the spleen with MACS columns (Miltenyi Biotech). Resting B cells were purified from spleen with B cell isolation kit (Miltenyi Biotech). Bone marrow derived dendritic cells were obtained by culture of bone marrow cells in RPMI1640 supplemented with 10% FCS for 7 days with human Flt3L (50 ng/ml). For transfections, cells were transiently transfected with Effectene (QIAGEN). To generate stable NIH3T3 cell transformants, cells were transfected with pCAG-PDLIM1 or empty vector and selected with G418 (500 mg/ml) for 14 days. For the reporter assay, 293T cells were transfected with the ELΑΜ−1 luciferase construct and expression plasmids encoding p65 and PDLIM1, or with the pGL4-NF-κB luciferase construct and expression plasmids encoding TRAF6, MyD88 or IKKβ and PDLIM1. Total amounts of transfected DNA were kept constant by supplementing with control plasmids. Luciferase activity was measured according to the manufacturer’s protocol in the Dual Luciferase Reporter System (Promega). Alternatively, MEF were transfected with the pGL4-NF-κB luciferase construct together with or without the PDLIM1 vector, then stimulated with LPS or poly (I:C) for 5 hr and luciferase activity was measured.

### Subcellular fractionation, immunoprecipitation and immunoblot analysis

All lysis buffers used for immunoblot analysis contained a proteinase inhibitor cocktail (complete mini; Roche). Cytoplasmic and nuclear fractions were prepared as follows. Cells were lysed on ice for 5 min with hypotonic buffer (20 mM HEPES, pH 8.0, 10 mM KCl, 1 mM MgCl_2_ 0.1% Triton X-100 and 20% glycerol). After centrifugation of samples at 5000 rpm for 1 min, supernatants were collected and were used as cytoplasmic fractions. Pellets were then lysed for 20 min on ice in hypertonic buffer (20 mM HEPES,pH 8.0, 1 mM EDTA, 20% glycerol, 0.1% Triton X-100 and 400 mM NaCl) with brief vortexing. After centrifugation at 15000 rpm for 5 min, supernatants were collected and used as nuclear fractions. The purity of the obtained fractions were confirmed with anti-HSP90, PKC or Cdc37 (for the cytoplasm) or anti-LSD1 (for the nuclear solution). Whole cell extracts were prepared by lysing cells in RIPA buffer (25 mM Tris pH 7.6, 150 mM NaCl, 1% NP-40, 1% sodium deoxycholate, 0.1% SDS). For immunoprecipitation in 293T cells (Fig. 2c), cells were transfected with plasmids encoding FLAG-p65 with or without c-Myc-PDLIM1. Extracts were prepared by lysing cells in 50 mM Tris 8.0, 0.5% NP-40, 5 mM EDTA, 50 mM NaCl, 50 mM NaF, incubated with anti-c-Myc antibody-conjugated agarose beads (MBL), washed four times and subjected to immunoblot analysis with the indicated antibodies. For immunoprecipitation in MEF and BMDC (Fig. 5b), cells were lysed in RIPA buffer and whole cell extracts were subjected to immunoprecipitation with control IgG or anti-PDLIM1 antibody followed by immnoblot with anti-α-actinin antibody.

### Ubiquitination assay

293T cells were transfected with expression plasmids encoding FLAG-tagged p65, histidine-tagged ubiquitin and c-Myc-PDLIM1 or PDLIM2, and His-tagged proteins were purified as previously described^33^. Briefly, transfected cells were extracted under denaturing condition with a buffer containing 6 M guanidium-HCl. Extracts were incubated with Ni-NTA beads (Clontech) for 2.5 hr and then washed with buffer containing 25 mM Tris pH 6.8, 20 mM imidazole. Purified proteins were subjected to immunoblot with anti-p65 or anti-c-Myc antibody.

### Immunofluorescence and confocal microscopy

MEF was seeded onto poly-L-lysine-coated slides, fixed for 15 min with 4%(w/v) paraformaldehyde and permeabilized for 10 min with 0.5%(v/v) Triton-X-100. Cells were blocked with 10% FBS in PBS and incubated for 1 hr with primary antibodies (1:100 in PBS) and for 1 hr with secondary antibodies (1:200 in PBS). For immunofluorescence with an *in situ* proximity-ligation assay (Fig. 1d), a Duolink *In situ* PLA Kit was used according to the manufacturer’s instructions (Olink Bioscience). Anti-PDLIM1, Duolink *In Situ* PLA probe anti-rabbit PLUS, Duolink *In Situ* PLA probe anti-rabbit MINUS and Duolink *In Situ* Detection Reagents Orange (Olink Bioscience) were used to detect PDLIM1 expression. Images were obtained with a Leica confocal TCS SP2 AOBS (Leica microsystems).

### Small interfering RNA (siRNA)

Lipofectamine RNAiMAX (Invitrogen) was used for the transfection of siRNA. The following siRNA were purchased from Invitrogen (Stealth RNAi); murine α-actinin-1, 5′-TGACGATGCTCTTCTCATACTGCCG-3′; murine α-actinin-4, 5′-TTCCGAAGATGAGAGTTGCACCAGG-3′; and control siRNA (12935–300). The siRNA specific for murine PDLIM1 (s79437), human IκBα (s9510), and control siRNA (4390843) were purchased from Ambion (Silencer Select).

### Real-time reverse transcriptase-polymerase chain reaction (RT-PCR) analysis

Total RNA was prepared using an RNAeasy micro kit (Qiagen) and cDNA was generated using a PrimeScript RT reagent kit (Takara Bio Inc.). Quantitative real-time PCR analyses were performed by TaqMan real-time PCR using a StepOnePlus (Applied Biosystems). The primer sets and probes for mouse *Il-6* (Mm00446190), *Il-12*p40 (Mm00434174), *Tnf*α (Mm00443258), *Il-18* (Mm00434225), *Cxcl2* (Mm00436450), *Cxcl10* (Mm00445235), *Mmp3* (Mm00440295), *Mmp9* (Mm00442991), *pdlim1* (Mm00834521) and 18S rRNA (4319413E) from TaqMan Gene Expression Assay (Applied Biosystems) were used for the reactions. To analyze CD83 expression, we used the SYBR Premix Ex Taq II kit (Takara Bio Inc.) and the primer pairs 5′-CGCAGCTCTCCTATGCAGTG-3′ and 5′-GTGTTTTGGATCGTCAGGGAATA-3′ (Invitrogen). Data were normalized to the amounts of 18S rRNA.

### Generation of *pdlim1*-deficient mice

Murine *pdlim1* genomic DNA was obtained by PCR (KOD plus DNA polymerase, TOYOBO) using C57BL/6 mice genomic DNA as a template. To construct the targeting vector, the neomycin phosphotransferase gene with a polyA signal derived from pMC1Neo-polyA (Stratagene), was inserted into exon 2 and the MC1-herpes simplex virus thymidine kinase (HSV-TK) was inserted in the 3′ end of homologous region. The targeting vector was electroporated into M1 ES cells, which were derived from F1 C57BL/6JJcl × 129 + Ter/SvJcl mice. Cells were cultured with G418 (Nacalai Tesque) and Ganciclovir (InvivoGen) and colonies resistant to both drugs were selected. Homologous recombinants were identified by PCR and this was subsequently confirmed by Southern blotting using the probe that can detect a *HindIII*-digested 11 kb fragment from the wild-type allele but a 5 kb fragment from the mutated allele. Targeted ES cells were aggregated with tetraploid embryos from BDF2 mice to generate chimeric mice. To obtain heterozygous mice, chimeric mice were bred to C57BL/6 mice and homozygotes were generated by intercrossing of heterozygotes. Mice were maintained under specific pathogen free conditions. All mice used were between 4 to 5 weeks of age. All experiments were approved by the RIKEN Yokohama Campus Animal Use Committee, and performed in accordance with the committee’s guidelines.

### Statistical Analyses

All of the *in vitro* experiments were repeated at least three times. Differences were analyzed by Student’s t-test. Data are presented as the mean values ± the standard deviation of the mean (SD).

## Additional Information

**How to cite this article**: Ono, R. *et al.* PDLIM1 inhibits NF-κB-mediated inflammatory signaling by sequestering the p65 subunit of NF-κB in the cytoplasm. *Sci. Rep.* **5**, 18327; doi: 10.1038/srep18327 (2015).
